# Clinical profile and predictors of Severe Dengue disease: A study from South India

**DOI:** 10.22088/cjim.9.4.334

**Published:** 2018

**Authors:** Vijay Kumar Agrawal, B. Saroj Kumar Prusty, Ch Santosh Reddy, Gangireddy Krishna Mohan Reddy, Rakesh Kumar Agrawal, Venkata Chandra Sekher Srinivasarao Bandaru

**Affiliations:** 1 Department of General Medicine, Yashoda Hospital, Hyderabad, India; 2 Department of Critical Care, Yashoda Hospital, Hyderabad, India; 3 Department of Research, Yashoda Hospital, Hyderabad, India

**Keywords:** Clinical manifestation, Laboratory test, Delayed admission, Severe dengue

## Abstract

**Background:**

Dengue is endemic and prevalent in tropical and sub-tropical countries including India and can cause significant mortality and morbidity. There are limited studies available on factors associated with severe dengue from India, to investigate the predictors of severe dengue in south Indian patients.

**Methods:**

We recruited 334 patients with dengue admitted in Yashoda Hospital, Hyderabad. Study period was between March 2015 and February 2017. Based on clinical symptoms, we divided patients into severe dengue and non-severe dengue. Univariate and multivariate analysis was performed for prognostic factors of severe dengue.

**Results:**

Out of 334 patients, there were 186(55.6%) males with mean age 30.3±14.3 39 years (age range: 10-73 years), severe dengue was seen in 117(35%) and non-severe dengue in 217(65%). Clinical symptoms of diabetes, low platelet count (<50,000mm^3^), melena, skin rash, delayed admission (>5days after onset) elevated hematocrit, lymphadenopathy, hepatomegaly, splenomegaly, convulsions and mortality were significantly associated with severe dengue. After multivariate analysis, diabetes (OR: 2.12; 95% CI:1.34-4.65) (<0.0001), elevated hematocrit (OR: 3.14; 95% CI:2.17-6.14) (<0.0001), skin rashes (OR: 1.99; 95% CI: 1.11-3.55) (<0.0001), melena (OR: 2.59; 95% CI:1.40-4.93) (<0.0001), low platelet count (OR: 6.71; 95% CI:4.12-13.6) (<0.0001), lymphadenopathy (OR: 3.12 95% CI: 1.91-7.85) (<0.0001) and delayed admission (OR: 2.40; 95% CI:1.31-3.41) (<0.0001) were significantly associated with severe dengue disease.

**Conclusions:**

In our study, it was established that low platelet count, elevated hematocrit, diabetes, skin rash, melena, lymphadenopathy and delayed in admission (>5days) were independently associated with severe dengue.

Dengue infection has become a common mosquito-borne viral disease, occurs in tropical and subtropical countries especially South and Southeast Asia countries, the Caribbean, Central and South America, and Africa (1). Worldwide every year, 50-200 million are affected with dengue infection, with over 20,000 dengue-related deaths and the incidence has risen 30 times during the past six decades (2). In India, dengue infection is seen all over the country including rural and urban areas (3). Dengue virus belongs to Arbovirus group, and infection is characterized by disease, headache, loss of appetite, arthralgia, rash, abdominal pain, nausea and vomiting (2). The complication of dengue disease was not treated properly, the mortality rate increased more than 20% (1). Recent studies have identified a subset with more complications and high mortality in severe dengue compared to non-severe dengue (4). 

The aim of the study was to investigate the clinical symptoms, laboratory findings and mortality in severe dengue. Very limited studies are available on this topic from Indian subcontinent.

## Methods

**Figure 1:**
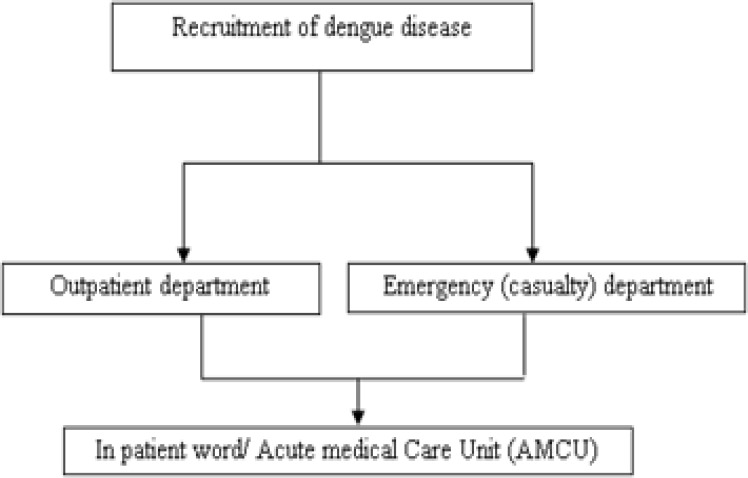
Design the flowchart of the patients’ entrance

We prospectively recruited 400 patients with primary presumptive diagnosis of dengue and were admitted in the Department of Medicine at Yashoda Hospital, Hyderabad (figure 1). Yashoda Hospital is a Post-graduate Teaching Hospital and a referral center of India, a study period between March 2015 and February 2017. Severe dengue was defined by World Health Organization (WHO) criteria (3, 5). This study was approved by the Institutional Ethics Committee (IEC) and consent was obtained from all the patients. 

Out of the 400 patients, 66 patients were excluded (twenty seven patients had incomplete data, and 39 patients left against medical advice (LAMA) within 3 days of admission), remaining 334 patients were included in the study. Patients underwent laboratory investigations including complete blood test, liver function tests, urine analysis (including urine albumin), abdominal ultrasound examination, serum creatinine, blood urea, serum albumin, serum glucose, cerebrospinal fluid analysis and chest x-ray reports were collected. 

In the present study, 120 (35.4%) had hepatic tenderness (35.9%), 14 (4.1%) with jaundice, 312(93.4%) had elevated liver enzyme, aspartate aminotransferase (AST) ranged 33-1082 U/L and 29-1028U/L for alanine aminotransferase (ALT).

 Out of 334 dengue patients, 270 had gallbladder (GB) wall thickening, 210 with pleural effusion, 179 had ascites, and 22(6.5%) subjects were found with splenomegaly and hepatomegaly in 26(7.7%) patients. In our patients, there were no history of disease like Japanese encephalitis virus (JEV), St. Louis encephalitis virus (SLE), West Nile virus (WNV) leptospirosis, yellow fever, malaria and hepatitis A, B, and C. Twenty (5.9%) patients had electroencephalogram. Thirty five (10.4%) patients had brain CT scan, forty patients (11.1%) underwent 2D echogram (26 patients had normal echocardiography, 10 had <45 of ejection fraction, two patients with global hypokinesia, one had pericardial effusion and one patient with left ventricular dysfunction), magnetic resonance imaging (MRI) was performed on eight patients (2.3%). 

**Serological examination: **In the present study, enzyme-linked immunosorbent assay (ELISA), specific to dengue, IgG and IgM antibody tests were performed in all patients (100%) while non-structural protein 1 (NS1) antigen test was performed in 140 (41.9%) patients. In our study, cutoff value of IgM ≤11, IgG ≤ 22 and NS1 antigen ≤ 11 were negative while IgM ≥12, IgG ≥ 23 and NS1 antigen ≥ 12 were considered positive. 

**Statistical analysis: **Statistical analysis was performed using SPSS Inc (Statistical Package for the Social Sciences) 16.0 software. Mean and standard deviation (SD) were calculated. The paired t-test was applied for differences in continuous variables. Univariate and multivariate analysis was performed for potential confounders (diabetics, elevated hematocrit, elevated PTT (>1.5) melena, skin rash, delayed in admission (>5days), and lymphadenopathy) for sever dengue. All tests were two-sided and a p-value <0.05 was considered statistically significant.

## Results

Out of 334 patients with dengue, 213(63.7%) were men with mean age of 30.3±14.3 years and age range of 10-73 years. Most common symptoms were fever seen in 100%, followed by myalgia in 88%, chills in 74.5% nausea/ vomiting 65.5% and headache in 38% of patients. On clinical examination were severe dengue in 35%, and non-severe dengue in 64.9%, convulsion was a rare complication seen in 4.1% of patients. Elevated hematocrit (>40%) was present in 41% and mean platelet count was 0.82±0.67 (table 1). Among the 334 patients with dengue, IgM positivity was seen in 203 (60.7%), IgG positivity in 73(21.8%), while 8.9% had both IgM and IgG antibodies positive and 140 (41.9%) were NS1 antigen test positive patients.

Delay in admission (>5 days onset) (p<0.0001), low platelet count (<50,000 mm^3^) (p<0.0001) melena (p<0.0001), skin rash (p<0.0001) lymphadenopathy (p<0.0001) elevated hematocrit (p<0.0001), elevated PTT(>1.5) (p=0.003), splenomegaly (p<0.0001), convulsion (p=0.02), hepatomegaly (p<0.0001), duration in hospital stay >5days and mortality (p=0.02) were significantly more prevalent in severe dengue compared to non-severe dengue. After multivariate analysis, we established that the major predictors of severe dengue were diabetes (OR: 2.12; 95% CI: 1.34-4.65), elevated hematocrit (OR: 3.14; 95% CI: 2.17-6.14), melena (OR: 2.59; 95% CI:1.40-4.93) skin rash (OR: 1.99; 95% CI: 1.11-3.55) delay in admission >5days) (OR: 2.40; 95% CI:1.31-3.41), low platelet count (<50,000 mm^3^) (OR: 6.71; 95% CI:4.12-13.6) headache (OR: 0.99; 95% CI:0.51-1.84) and lymphadenopathy (OR: 3.12 95% CI: 1.91-7.85) (Table 2).

**Table 1:** Comparison of basal characteristics between severe dengue and non-severe dengue

	**Severe dengue** **(includes deaths)** **(n=117)**	**Non-severe dengue** **(n=217)**	**Total**	**P value**
Men	66(56.4%)	120(55.2%)	186 (55.6%)	0.8
Women	51(43.5%)	97(44.7%)	148 (44.4%)	0.8
Mean age (years)	30.4±11.8	30.3±15.4	30.3±14.3	0.9
Hypertensive	15(12.8%)	18(8.2%)	33 (9.8%)	0.4
Diabetes	45(38.4%)	37(17%)	82 (24.5%)	0.001
Delay in admission (>5days )	43(36.7%)	31(14.2%)	74 (22.1%)	0.0001
Low platelet count(<50,000 mm^3^)	69(58.9%)	23(10.5%)	92 (28.7%)	0.0001
Chills	79(67.5%)	170(78.3%)	249 (74.5%)	0.1
Headache	51(43.5%)	76(35%)	127 (38%)	0.0001
Myalgia	97(82.9%)	197(90.7%)	294 (88%)	0.5
Arthralgia	49(41.8%)	63(29.8%)	112 (33.5%)	0.09
Melena	40(34.1%)	33(15.2%)	73 (21.8%)	0.0001
Rashes	53(45.2%)	50(23%)	103 (30.8%)	0.0001
Nausea and vomiting	82(70%)	137(63.1%)	219 (65.5%)	0.6
Abdominal Pain	71(60.6%)	110(50.6%)	181 (54%)	0.1
Lymphadenopathy	27(23%)	13(5.9%)	40 (11.9%)	0.0001
Fever >39^0^C	48(41%)	92(42.3%)	140 (41.9%)	0.8
Elevated hematocrit (>40%)	76(64.9%)	61(28.1%)	137 (41%)	0.0001
Hypotension	18(15.3%)	20(9.2%)	38 (11.3%)	0.1
Bradycardia	12(10.2%)	0	12 (3.5%)	0.0001
Elevated Plasma Thromboplastin Time (>1.5)	58(49.5%)	67(30.8%)	125 (37.4%)	003
Splenomegaly	22(18.8%)	0	22 (6.5%)	0.0001
Convulsions	14(11.9%)	0	14 (4.1%)	0.02
Hepatomegaly	26(22.2%)	0	26 (7.7%)	0.0001
Deaths	5(4.2%)	0	5 (1.4%)	0.02

**Table 2:** Predictors of severe dengue

	**Univariate**		**Multivariate**		**p value**
	**Odds ratio**	**95% CI**	**Odds ratio**	**95% CI**	
Diabetes	3.04	1.81-5.08	2.12	1.34-4.65	<0.0001
Elevated hematocrit (>40%)	4.74	2.92-7.67	3.14	2.17-6.14	<0.0001
Elevated Plasma Thromboplastin Time (>1.5)	2.20	1.38-3.49	0.91	0.51-1.92	<0.03
Skin rash	2.76	1.70-4.47	1.99	1.11-3.55	<0.0001
Melena	3.26	1.72-6.18	2.59	1.40-4.93	<0.0001
Low platelet count	12.1	6.87-23.3	6.71	4.12-13.6	<0.0001
Headache	1.45	0.93-2.34	0.99	0.51-1.84	0.04
Abdominal pain	2.01	1.27-3.19	0.8	0.05-1.91	0.02
Lymphadenopathy	4.70	2.32-9.54	3.12	1.91-7.85	<0.0001
Delay in admission (>5days)	2.91	1.82-4.66	2.40	1.31-3.41	<0.0001

## Discussion

In our study, we established 35% of severe dengue, other studies found similar findings (6-12). Ledika et al. noted 24.6% in their studies (6), Aung et al. 27.9% (7), Mena-Lora et al. 26% (8) of patients with severe dengue. Some studies showed the lower prevalence of severe dengue at 9.0% (9). The present study revealed no significant association between age and severe dengue when dichotomized into <40 years and above 40 years of age, these findings were advocated by others (10). Nonetheless, few studies have shown a significant association with young age group (8) and older age group (11) with severe dengue.

There is contradictory evidence regarding the association of gender with severity of dengue. In our study, we found no significant association of gender with severe dengue (men 56.4% vs women 43.5%), the similar findings were noted by others (10). In contrast, few studies found significant association with female gender (12), while others showed men to be more prone to develop severe dengue (11). Many studies have shown diabetes to be a comorbid factor for severe dengue (13).

 In a study by Saqib et al., 60% of diabetes cases had severe dengue (14). In the current study, we established independent association of diabetes with severe dengue (odds 2.12; 95% CI:1.34-4.65), our finding was supported by others (15). The pathophysiology behind diabetes leading to severe organ involvement outcome is not well understood yet. The innate immunity is already altered in diabetes mellitus and results in an existing pro-inflammatory state with endothelial dysfunction. Co-occurrence of dengue infection can further worsen the condition with cytokine overload, which exaggerates the endothelial damage causing vascular leak and dengue hemorrhagic disease predisposing to severe dengue (15). Diabetes may also worsen the thrombocytopenia in patients with dengue, thus, adding its contribution to the development of severe dengue (16).

The presence of abdominal pain the initial manifestation of dengue is now well recognized (4, 17). In our study, we found a prevalence of abdominal pain in patients with dengue 181 (54.1%) (71 with severe dengue and 94 with non-severe dengue), similar findings were recommended by others (17). Our study established no significant association with severe dengue, a similar finding was noted by Mena Lora et al. (8). Nevertheless, Zhang et al. found an independent association with abdominal pain (odds: 2.278; 95% CI: 1.631, 3.182) (4). Hepatomegaly, as a warning indication for severe dengue has been suggested by WHO (6). In addition, our study found hepatomegaly in 26 (22.2%) patients with severe dengue compared to no one in non-severe dengue, suggesting a high positive predictive value (100%) of hepatomegaly in detecting severe dengue, similarly a strong association and an independent predictive value of hepatomegaly has been advised by others (6). 

Splenomegaly may occur due to viral infection inducing inflammatory immune response in patients with dengue and showed a strong association with severe dengue (18) while others did not (19). The current study showed splenomegaly only in patients with severe dengue 22(18.8%) (p<0.0001), adding to the data supporting a significant association with severe dengue. The most prevalent manifestation was melena in this cohort. Seventy three (21.9%) cases of dengue had melena and previous studies showed comparable outcome (4, 20-23). In the studies of Mohan et al. Laul et al and Mandal et al. a prevalence of 19% (20), 26% (21) and 26.8% respectively they reported (22). In the present study, we identified a significantly higher prevalence of melena among severe dengue patients (34.1%). On multivariate analysis, we found an independent association with severe dengue (odds: 2.59; 95% CI: 1.40-4.93), our findings were supported by other researchers (4). 

Skin rashes are frequently present in dengue disease and the present study established an independent association with severe dengue (OR: 1.99; 95% CI:1.11-3.55). Our finding was supported by Zhang et al. (odds: 2.03; 95% CI:1.26-3.25) (4) and Khan et al. (OR;9.16; 95% CI:4.04- 20.78) (24), while few studies showed no association (23). An immunological mechanism may be the explanation for developing these rashes. Dengue virus can incite the production of cytokines with stimulation of vascular endothelial changes, infiltration of mononuclear cells and perivascular edema, consequently leading to a skin rash (25). The present study showed 23% in lymphadenopathy with severe dengue and the previous studies have noted lymphadenopathy in 5-40% of patients with severe dengue (26). In our study showed lymphadenopathy was found to be an independent predictor of severe dengue (OR: 3.12; 95% CI: 1.91-7.85). Headache and retro-orbital pain are well-established symptoms in dengue (26) and are present in 60-90% of cases with dengue (22). Our study showed no significant association of headache with severe dengue. These findings were supported by other researchers (4). Central nervous system (CNS) features are rare but can occur in dengue and in our study, we found convulsion in 14 (11.9%) patients who were significantly associated with severe dengue, our findings were advocated by others (10). Seizures can be associated with encephalopathy, hemorrhages, infarction or metabolic disorders the may effect dengue encephalitis (27) or secondary to immunological mechanism (28). 

Elevated hematocrit suggests a vasculopathy along with leakage, secondary to increasing vascular permeability is an indirect measure of the cytokines being produced and the possibility of severe vascular endothelial dysfunction and usually predate shock. Recent studies have established hematocrit > 40% as a prognostic factor for severe dengue (28). Additionally, in our study, hematocrit (≥40%) emerged as a strong independent predictor of severe dengue (OR: 3.14; 95% CI: 2.17-6.14). Nonetheless, a recent study has not found any association between elevated hematocrit and severe dengue infection in children (29). Thrombocytopenia is one of the potential indicators of severe dengue (6). In our study, platelet count ≤50,000/ mm3 was significantly higher in patients with severe dengue (58.9%) compared to non-severe dengue (10.5%) (p<0.000). 

The exact pathophysiology of thrombocytopenia in dengue is not yet clearly elucidated. Dengue virus may have a direct effect on the bone marrow -specially the progenitor cells causing a reduction in their capacity to replicate. An aberrant immunological response, implicated in severe dengue seems to play a significant role by dysregulation of plasma-kinin system. This leads to an increased consumption of platelets by disseminated intravascular coagulation (DIC). The damage is enhanced by increased apoptosis of platelets and generation of antiplatelet antibodies (6). Our study further emphasizes low platelet count (≤50,000/ mm3) as an independent factor for severe dengue (OR: 6.71; 95% CI: 4.12-13.6). Admission delay was one of the major risk factor for severe dengue which may have contributed to death. The present study noted delay in admission (≥5days onset) was significantly higher among the patients with severe dengue 43(36.7%) (p<0.0001), our study was advocated by others (6, 17). Ledika et al. (6) in his study noted a delay of more than 4 days of onset was significantly associated with severe dengue. In our study, we established a delay in admission as an independent predictor of severe dengue (OR: 2.40; 95% CI:1.31-3.41), these findings were supported by other researchers (6, 12). Delay in admission in severe cases may be due to the administrative issues such as lack of knowledge, misdiagnosis or lack of funds. Besides, the immunological dysfunction may manifest a couple of days after the viral infection itself, resulting in a severe disease, eventually getting medical assistance. 

Studies have established the mortality rate of 1-4% in severe dengue (30). The present study demonstrated a mortality rate of 2.4%. Mortality was significantly associated with severe dengue in our study and there was no mortality among non-severe dengue patients. Similar studies found a case fatality note of 1.2% in severe dengue (6, 7). Severity is defined as having more risk of mortality associated with severe bleeding, plasma leakage, shock and other organ involvement (5). In our study, we found 5 patients who died with elevated transaminase levels and delayed admission (>5 days). Three patients had severe bleeding, two with convulsions, three patients had concomitant diabetes and hypertension. The present study has few limitations: we were unable to analyze seasonal variation, rural and urban area and quantitative analysis of levels of IgM and IgG antibodies against dengue or the glycemic control in diabetic patients with severe dengue. The strengths of our study was conducted at a single center and a single laboratory to confirm dengue. In addition, this study followed current WHO guidelines for severe dengue and we did multiple regression analysis for predictors of severe dengue. 

In the current study in South Indian population, we established that elevated hematocrit (>40%), low platelet count (<50,000mm^3^), diabetes, skin rash, melena, lymphadenopathy and delay in admission (>5days) were independently associated with severe dengue disease. The was a low mortality rate (4.2%) in our cohort. Further studies to compare the strength of association of these factors can help in creating an algorithm for predicting the occurrence of severe dengue.
